# Preparation of Protein A Membrane Adsorbers Using Strain-Promoted, Copper-Free Dibenzocyclooctyne (DBCO)-Azide Click Chemistry

**DOI:** 10.3390/membranes13100824

**Published:** 2023-10-06

**Authors:** Joshua Osuofa, Scott M. Husson

**Affiliations:** Department of Chemical and Biomolecular Engineering, Clemson University, Clemson, SC 29634, USA

**Keywords:** Protein A, membrane chromatography, alkyne–azide, copper-free click chemistry, antibody purification

## Abstract

Protein A chromatography is the preferred unit operation for purifying Fc-based proteins. Convective chromatography technologies, like membrane adsorbers, can perform the purification rapidly and improve throughput dramatically. While the literature reports the preparation of Protein A membrane adsorbers utilizing traditional coupling chemistries that target lysine or thiol groups on the Protein A ligand, this study demonstrates a new approach utilizing copper-free dibenzocyclooctyne (DBCO)-azide click chemistry. The synthetic pathway consists of three main steps: bioconjugation of Protein A with a DBCO-polyethylene glycol (PEG) linker, preparation of an azide-functionalized membrane surface, and click reaction of DBCO-Protein A onto the membrane surface. Using polyclonal human immunoglobulins (hIgG) as the target molecule, Protein A membranes prepared by this synthetic pathway showed a flowrate-independent dynamic binding capacity of ~10 mg/mL membrane at 10% breakthrough. Fitting of static binding capacity measurements to the Langmuir adsorption isotherm showed a maximum binding (q_max_) of 27.48 ± 1.31 mg/mL and an apparent equilibrium dissociation constant (K_d_) of value of 1.72 × 10^−1^ ± 4.03 × 10^−2^ mg/mL. This work represents a new application for copper-less click chemistry in the membrane chromatography space and outlines a synthetic pathway that can be followed for immobilization of other ligands.

## 1. Introduction

Click chemistry is versatile and has been lauded for bio-based applications due to high specificity, high fidelity (i.e., no side products), mild reaction conditions, and its ability to operate at low concentrations. The term “click” chemistry originally was coined to describe the Cu(I)-catalyzed alkyne–azide reaction chemistry developed by Sharpless and coworkers in 2002 [1]. Since then, it has been utilized widely in the fields of chemical biology for many applications, including preparation of antibody-drug conjugates and vaccines; imaging and profiling of all classes of biomolecules including but not limited to proteins, glycans, lipids, metabolites; immobilization of biomolecules onto stationary supports; and the development of new classes of biorthogonal chemistry like tetrazine ligation chemistry [2,3]. While Cu(I) alkyne–azide chemistry has been used to prepare chromatography media [4,5,6,7], there have been no instances in the literature where it has been used specifically for preparation of Protein A chromatography media.

While Cu(I)-catalyzed alkyne–azide click chemistry remains popular for the reasons mentioned above, the requirement to use copper is a significant drawback because it is cytotoxic, and for development of chromatographic supports, it might bind to the surface of the chromatography medium during and after ligand immobilization [5]. Strain-promoted click chemistry was developed to overcome these copper-related issues. Bertozzi and coworkers [8] found that the same benefits of Cu(I) alkyne–azide click chemistry could be realized without using copper by replacing the terminal alkyne with an internal alkyne within a ring structure. This class of compounds, termed cyclooctynes, undergo cycloaddition with azides under physiological conditions, with the ring strain acting as the catalyst instead of Cu(I). One of the most applied uses of strain-promoted click chemistry has been for reactions in living systems [8,9]. In this study, the main interest was to use this chemistry for covalent protein immobilization onto a chromatography support.

In the field of protein immobilization onto solid supports, immobilization chemistry plays a major role in immobilization yield, activity of immobilized protein, and performance of the solid support. In a recent study by Zhu and Sun [10], multiple chemistries were evaluated for Protein A/G immobilization onto poly(vinyl alcohol-*co*-ethylene) nanofibers under similar conditions. These chemistries included: epibromohydrin (EP), 1,4-butanediol diglycidyl ether (BDGE), oxalyl chloride (OA), nitrophenyl chloroformate (NP), cyanuric chloride (CC), carbonyldiimidazole (CDI), gluteraldehyde (GA), and disuccinimidyl carbonate (DSC). Of these chemistries, GA and DSC resulted in the highest activities and immobilization yields.

Based on the strong affinity of Protein A to the Fc-region of antibodies, Protein A membrane adsorbers can be used for the rapid purification of antibodies and other Fc-fusion proteins [11]. Previous attempts to develop Protein A membrane adsorbers have used other coupling chemistries such as EDC/NHS (1-ethyl-3-(3-dimethylaminopropyl)carbodiimide/N-hydroxysuccinimide) [12], EDC/Sulfo-NHS [13], cyanogen bromide [14], and GA [10]. To our knowledge, there has been no attempt at preparing Protein A membrane adsorbers using strain-promoted click chemistry. We therefore present a new approach for their preparation via dibenzocyclooctyne (DBCO)-azide strain-promoted click immobilization of Protein A onto regenerated cellulose membrane supports. The synthetic approach includes three main steps: incorporation of DBCO groups into Protein A via NHS ester bioconjugation; preparation of azide functionalized membranes using the DSC functionalization of hydroxyl groups on the membrane supports, followed by reaction with an azido-PEG_3_-amine molecule; and finally, a click reaction between DBCO-conjugated Protein A and the azide-functionalized membrane surface.

## 2. Materials and Methods

### 2.1. Materials

The following materials were purchased from MilliporeSigma: 3-azido-1-propanol (>96%), 4-dimethylaminopyridine (DMAP, >99%), acetonitrile (HPLC grade, >99.9%), alpha-cyano-4-hydroxycinnamic acid (α-CHCA, 99%), copper(II) sulfate pentahydrate (CuSO_4_-5H_2_O, >98.0%), dimethyl sulfoxide (anhydrous, >99.9%), dimethylformamide (anhydrous, >99.8%), Folin–Ciocalteu’s phenol reagent (F&C reagent, product no. 47641), formic acid (reagent grade, >95%), guanidine hydrochloride (>99%), hydrochloric acid (HCl, 37%), potassium sodium tartrate tetrahydrate (>99%), phosphate-buffered saline (PBS, powder, product number P3813), sodium carbonate (Na_2_CO_3_, >99.5%), sodium chloride (NaCl, 98%), sodium dodecyl sulfate (SDS, >98%), sodium phosphate dibasic (>99%), trisbase (>99.7%), N,N′-disuccinimidyl carbonate (DSC, >95%), Whatman regenerated cellulose membrane filters (RC-58, diameter 47 mm, pore size 0.2 µm).

Polyclonal Immunoglobulin G (IgG) from human plasma was purchased from Lee Biosolutions (Product number 340-21, >95%). Native recombinant Staphylococcal Protein A ligand was purchased from Repligen (rSPA, Product number 10-2001-0M). DBCO-PEG_5_-NHS ester was purchased from Click Chemistry Tools (Catalog number A102P-100, >95% by HPLC). Azido-PEG_3_-amine was purchased from BroadPharm (Catalog number BP20580, 98%).

### 2.2. Preparation of DBCO-Conjugated Protein A

#### 2.2.1. Conjugation with DBCO-PEG_5_-NHS Ester

DBCO-PEG_5_-NHS ester reacted with Protein A at different molar ratios. The following procedure outlines the preparation using a 15 to 1 molar ratio with respect to Protein A. Protein A stock solution was supplied by Repligen at 50 mg/mL in a sodium chloride buffer solution. A 50 mM stock DBCO-PEG_5_-NHS ester solution was prepared in anhydrous DMSO. One milliliter of 50 mg/mL Protein A was diluted with 3.6 mL of 140 mM sodium phosphate buffer at pH 8.4. For a 15 to 1 molar ratio, 321 µL of the 50 mM DBCO-PEG_5_-NHS ester solution was added drop wise to the Protein A solution while gently mixing with a stir bar. The resulting Protein A was 10 mg/mL in 100 mM phosphate buffer at pH 8.3, and the reaction was performed for 12 h at room temperature.

After bioconjugation, excess DBCO reagent was removed using size exclusion chromatography. One milliliter of 10 mg/mL DBCO-Protein A was injected into Cytiva Superdex 75 10/300 GL at 0.4 mL/min. The mobile phase was 100 mM sodium phosphate at pH 8.3. Figure S1, in Supporting Information, shows the resulting chromatograms. The first peak of the chromatogram, spanning 5 mL in elution volume, corresponds to DBCO-Protein A and was collected for further use and analysis.

#### 2.2.2. Matrix Assisted Laser Desorption Ionization-Time of Flight (MALDI-TOF) Mass Spectrometry

MALDI-TOF mass spectrometry was performed to determine the success of the conjugation reaction. The matrix was prepared with 20 mg/mL α-CHCA in a 70/30 (*v*/*v*) ACN:5% formic acid (aq.) mixture. The plating procedure was as follows: 1 µL of matrix was plated and then allowed to dry. Then, 1 µL of 2 mg/mL Protein A solution was plated. Finally, 1.5 µL of matrix was added. All samples were analyzed using a Bruker Microflex^TM^ LRF MALDI-TOF in linear mode (105 cm flight path). Laser level was between 20 and 70%, detector gain was 2827 V, and the analysis used positive ion spectrum. The same procedure was carried out for DBCO-Protein A. Reported MW/charge (M/Z) values are the average of two peak measurements. The standard deviation of these measurement was reported as the error bars.

### 2.3. Membrane Surface Modification

**Scheme 1:**
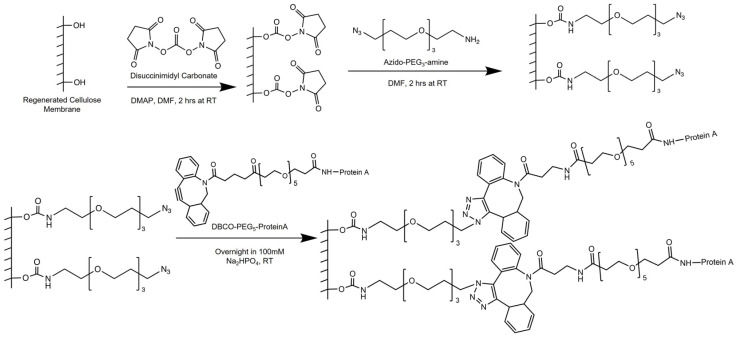
Synthetic pathway for Protein A membranes: DSC modified surface chemistry, azido-PEG_3_-amine reaction, and DBCO-conjugated Protein A immobilization.

Scheme 1 illustrates the functionalization procedure of the regenerated cellulose (RC-58) substrates. First, activated NHS ester molecules are created by reacting the hydroxyl groups with DSC. Activated NHS ester molecules undergo nucleophilic attack by primary amines of the azido-PEG_3_-amine molecules. The resulting scaffold is a membrane surface modified by azide moieties. DBCO-PEG_5_-conjugated Protein A is then clicked onto the azide groups of the membrane. A DBCO-PEG_5_-conjugated Protein A, prepared using a 30 to 1 molar ratio of DBCO-PEG_5_-NHS ester to Protein A, was used for the click reaction. The volumes used per membrane sample and final solution concentration of each substance are provided in the sections that follow.

#### 2.3.1. DSC Functionalization of Regenerated Cellulose Membranes

DMAP (579 mM, 0.991 g) was added to dry DMF (14 mL) and the solution was sonicated until DMAP dissolved fully. The RC-58 membrane was rinsed by immersing in 10 mL of dry DMF for 10 min. After rinsing, the membrane was soaked in the DMAP/DMF solution for a few minutes. DSC (343 mM, 1.232 g) was added, and the solution was placed on a shaker at 120 rpm. The solution turned brown as the reaction proceeded at room temperature for 2 h. After the reaction, the membrane was rinsed with DMF, DMSO, and 2-propanol. Functionalized membranes were stored in 2-propanol until the next reaction step.

#### 2.3.2. Azide Functionalization of Regenerated Cellulose Membranes

Azide-PEG_3_-amine (500 mM, 0.3 mL) was added into DMF (2.7 mL). A DSC-modified RC-58 membrane was immersed into the solution and reacted for at least 2 h at room temperature. Unreacted DSC groups were quenched using the following rinsing procedure at room temperature: (i) 1 M Trisbase, pH 9.1 (30 min); (ii) water (1 min, twice); (iii) 6 M guanidine, pH 7 (30 min); (iv) 1 M NaCl (10 min, three times). After the rinsing procedure, membranes were stored in water until the next step.

#### 2.3.3. Click Reaction with Azide Functionalized Membrane

An azide-modified RC-58 membrane was immersed into 2.5 mL of 100 mM PBS solution containing 10 mg/mL DBCO-conjugated Protein A. The solution was placed on a shaker at 120 rpm. The reaction was performed at room temperature for 8 h.

### 2.4. Characterization of Modified Membranes

#### 2.4.1. Attenuated Total Reflectance Fourier-Transform Infrared (ATR-FTIR) Spectroscopy

ATR-FTIR spectroscopy was used to analyze the surface chemistry of RC-58 membranes after each reaction step: DSC modification, azido-PEG_3_-amine attachment, and DBCO-Protein A click reaction. A Perkin Elmer Spectrum Two FTIR was used for analysis of all samples. The instrument was equipped with a Universal ATR accessory using a single reflection diamond crystal, and each spectrum was obtained using 32 scans at a resolution of 4 cm^−1^. Background correction, baseline correction, ATR-FTIR correction, and peak analysis were performed using PerkinElmer Spectrum 10 software.

#### 2.4.2. X-ray Photoelectron Spectroscopy (XPS)

XPS measurements were performed using a Physical Electronics PHI 5000 Versa Probe III Scanning ESCA Microprobe (Chanhassen, MN, USA). The instrument featured a 180° hemispherical electron energy analyzer and a monochromatic Al Kα scanning microprobe X-ray source that used a 15 kV beam volage and 100 µm spot size. High resolution scans were performed for C(1s), N(1s), and O(1s) using a step size of 0.125 eV and pass energy of 69 eV. Measurements were made under vacuum at <1.3 × 10^−6^ Pa.

#### 2.4.3. Ligand Density

Ligand density measurements were performed using a Lowry Assay. Stock solution A was prepared comprising 1/1/100 (*v*/*v*/*v*) aqueous mixture of 1 wt% copper(II) sulfate pentahydrate: 2.7 wt% potassium sodium tartrate: 2 wt% sodium carbonate. All solutions were prepared using DI water. Stock solution B was prepared comprising 1/1 (*v*/*v*) F&C reagent:DI water. One hundred microliter Protein A solutions were prepared at concentrations ranging from 0.05 to 1 mg/mL in small test tubes. The same was conducted for DBCO-Protein A samples. These samples were used as calibration standards for calculating the amount of covalently immobilized Protein A on the membranes. Protein A clicked membranes were cut into 5 mm diameter coupons and inserted into test tubes containing 100 µL of water.

To begin the assay, 1 mL of solution A was added to each tube, vortexed and incubated at room temperature for 10 min. One hundred microliters of solution B were added to each tube, vortexed and incubated at room temperature for 20 min. Three hundred microliters from each test tube were aliquoted into a clear 96 well plate and absorbance values were measured at a wavelength of 750 nm. The 750 nm absorbance readings of samples from test tubes containing DBCO-Protein A clicked membranes were converted to masses of DBCO-Protein A using the calibration standards. The mass values were divided by membrane volume to determine ligand densities in units of mg/mL membrane.

### 2.5. Performance of Protein A Membranes

#### 2.5.1. Static Binding Capacity (SBC)

SBCs of clicked Protein A membranes were measured using hIgG. A typical procedure for this experiment is as follows: using 1× PBS at pH 7.4 as the buffer solution, hIgG solutions were prepared at initial concentrations ranging from 0.1 to 5 mg/mL. One 2.5 cm diameter membrane was placed into a glass vial containing 2 mL of hIgG solution at a known concentration. The glass vial was put in a shaker bath at room temperature and 100 rpm for at least 12 h until equilibrium was reached. The equilibrium hIgG concentration was measured with a Nanodrop™ One Microvolume UV-Vis Spectrophotometer at 280 nm. A mass balance was performed using the initial (C_o_) and equilibrium (C_eq_) hIgG concentrations, the solution volume (V_sol_) and the membrane volume (V_mem_) to determine SBC, q (in mg hIgG adsorbed per mL of membrane) (Equation (1)). Each measurement was repeated in duplicate. Thermodynamic parameters, q_max_ and apparent K_d_, were determined by fitting SBC values using the Langmuir adsorption isotherm model (Equation (2)).

SBC = q = (C_o_ − C_eq_) × V_sol_/V_mem_ (1)

q = q_max_ × C_eq_/(K_d_ + C_eq_) (2)

#### 2.5.2. Dynamic Binding Capacity (DBC_10_)

DBC_10_ measurements were performed using an AKTA Pure 25 from Cytiva. Ten DBCO-Protein A clicked membranes, 23 mm diameter each, were stacked into a membrane holder in an axial format. Bind and elute studies were performed using hIgG solutions prepared by dissolving hIgG in the loading buffer (1× PBS, pH 7.4) and subsequently filtering through a 0.2 µm cellulose acetate syringe filter prior to use. DBC_10_ values were calculated at 10% breakthrough using Equation (3) where V_break_ is the effluent volume (mL) at 10% breakthrough, V_dead_ is the dead volume of the system (mL), C_o_ is the feed concentration (mg/mL), and V_mem_ is the volume of the membrane adsorber (mL).

DBC_10_ = (V_break_ − V_dead_) × C_o_/V_mem_ (3)

## 3. Results

### 3.1. Conjugation of Protein A Molecule in DBCO-PEG_5_-NHS Ester

Protein A contains five Fc-binding domains (namely E, D, A, B and C) and one lysine-rich domain (X domain). The protein contains 55 lysine residues but 30 of those reside in the X domain, while the other 25 are spread across the other five Fc-binding domains. In this study, the conjugation reaction with DBCO-PEG_5_ linker utilizes NHS ester chemistry which targets lysine residues via nucleophilic addition [15]. Increasing molar ratios of DBCO-PEG_5_-NHS ester to Protein A were investigated and MALDI-TOF mass spectrometry was used to analyze the molecular weights of the resulting conjugates.

**Figure 1:**
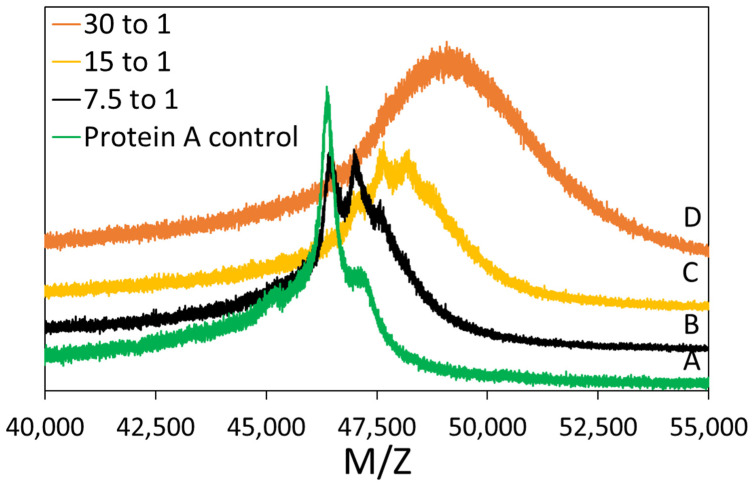
MALDI-TOF mass spectra of conjugated Protein A versus control Protein A. Protein A was conjugated with DBCO-PEG_5_-NHS ester at three in-solution molar ratios of DBCO-PEG_5_-NHS to Protein A: (A) unconjugated Protein A, (B) 7.5 to 1, (C)15 to 1 and (D) 30 to 1.

Figure 1 shows that there is a shift toward higher molecular weights as the molar ratio of DBCO-PEG_5_-NHS ester to Protein A in solution increases. This shift suggests successful incorporation of DBCO-PEG_5_ molecules into Protein A molecules and shows that more DBCO-PEG_5_ molecules are incorporated as the molar ratio in solution increases.

Analysis of the spectra at 7.5 to 1 and 15 to 1 conjugation ratios indicates that there are multiple peaks present. The molecular weight difference between the peaks is ~578 g/mol, which matches the molecular weight of the DBCO-PEG_5_-NHS ester linker. This suggests the presence of multiple Protein A species. In the case of 7.5 to 1 conjugation ratio, one peak corresponds to native Protein A, and the other peak corresponds to Protein A conjugated with one DBCO-PEG_5_ moiety. In the case of 15 to 1 conjugation ratio, multiple peaks correspond to Protein A conjugated with two and three DBCO-PEG_5_ moieties. For the 30 to 1 conjugation ratio, the broadness of the peak may indicate the presence of multiply functionalized species but the peak maximum indicates the highest increase in molecular weight is achieved. This result suggests that a 30 to 1 conjugation ratio leads to a maximum number of DBCO-PEG_5_ moieties. The 30 to 1 conjugation ratio was chosen to prepare DBCO-PEG_5_-Protein A conjugates used for membrane synthesis. Peaks corresponding to the higher molecular weight were used for calculating the number of incorporated DBCO-PEG_5_ molecules shown in Table 1.

### 3.2. Characterization of Modified Membranes

**Figure 2:**
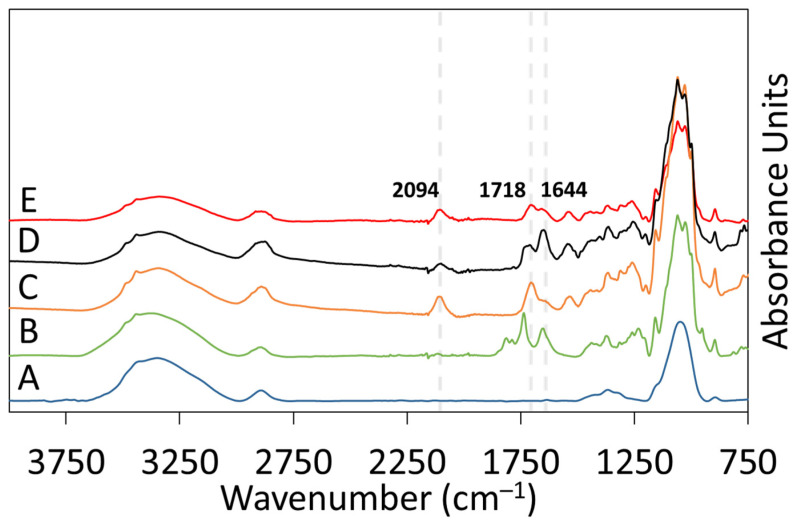
TR-FTIR spectra: (A) Unmodified membrane, (B) DSC-modified, (C) azido-PEG_3_-amine reacted on DSC modified membrane (D) DBCO-PEG_5_-NHS ester reacted with azido-modified membranes, (E) DBCO-Protein A reacted with azido-modified membranes.

ATR-FTIR was used to support the success of each consecutive reaction, as shown in Figure 2. Unmodified membranes were analyzed as a control (spectrum A). After DSC-modification, the spectrum (B) had new peaks: symmetric carbonyl stretching of the cyclic imide of the succinimide group at 1784 cm^−1^ and 1806 cm^−1^, carbonyl stretching of the carbonate group at 1732 cm^−1^, and amide stretching of NHS ester at 1644 cm^−1^. Reaction of NHS ester with azido-PEG_3_-amine results in disappearance of the peaks assigned to the NHS carbonyl group and carbonate, and the appearance of peaks assigned to the amide group stretching at 1698 cm^−1^, azide stretching at 2094 cm^−1^, and CH_2_ scissoring at 1558 cm^−1^ (spectrum C). Similar spectra have been reported for amine functionalized DSC surfaces [16].

Testing of the reactivity of the azide on the membrane surface was performed by a reaction with DBCO-PEG_5_-NHS ester alone. The resulting spectrum (D) is shown in Figure 2 and is characterized by the near-complete disappearance of the azide peak at 2094 cm^−1^, and the appearance of carbonyl and amide stretching peaks at 1718 cm^−1^ and 1644 cm^−1^. These two new peaks are characteristic of the NHS ester and DBCO molecules, and represent a successful reaction with the azide moiety on the membrane surface. Lastly, DBCO-Protein A reacted onto the surface of the azide functionalized membrane. The resulting spectrum (E) is similar to that of the DBCO-PEG_5_ reacted membranes, with the exception that the azide peak at 2094 cm^−1^ does not disappear completely. This result suggests that not all azide moieties are reacted, likely due to steric hindrance caused by the large size of DBCO-Protein A molecules.

To further characterize the membrane surface after reaction with DBCO-Protein A, XPS was used to measure and compare the atomic content of the azido-PEG_3_-amine surface with that of the DBCO-Protein A reacted surface. Table 2 compares the atomic compositions. Notably, there is an increase in nitrogen content from 6.34 ± 0.06% to 10.45 ± 0.04%. This increase is attributed to the presence of immobilized Protein A.

### 3.3. Performance of Clicked Protein A Membranes: Static (Equilibrium) and Dynamic Binding Capacity

#### 3.3.1. Static Binding Capacity (SBC)

**Figure 3:**
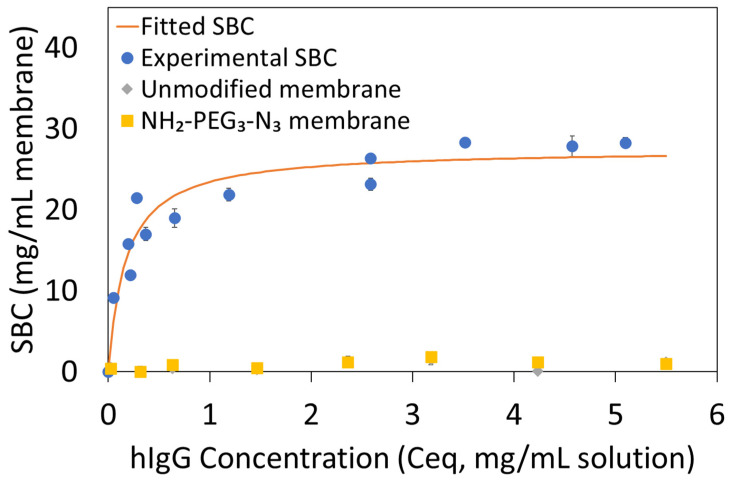
Static binding capacity measurements. Blue symbols represent experimental data for DBCO-PEG_5_-Protein A modified membranes, and the curve represents the best fit to the Langmuir adsorption isotherm model.

SBC (also known as equilibrium binding capacity, EBC) measurements for hIgG were performed using batch contact with membranes. hIgG thermodynamic adsorption parameters were calculated by fitting SBC values to the Langmuir adsorption isotherm model. Figure 3 shows the room temperature adsorption isotherm. q_max_ and apparent K_d_ were calculated to be 27.48 ± 1.31 mg/mL membrane and 1.72 × 10^−1^ ± 4.03 × 10^−2^ mg/mL solution.

#### 3.3.2. Dynamic Binding Capacity (DBC_10_)

**Figure 4:**
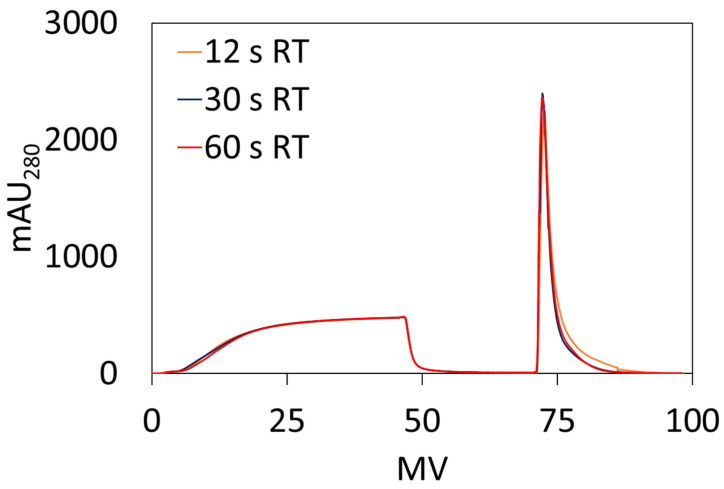
Chromatograms for hIgG at three residence times using a chromatography column packed with 10 stacked membranes. Membranes were prepared using a DBCO-PEG_5_-NHS ester to Protein A molar ratio of 30 to 1 in solution.

**Figure 5:**
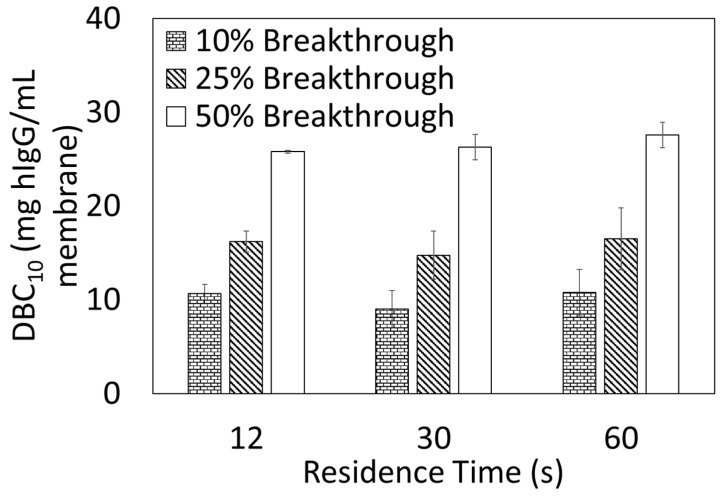
Calculated DBC_10_ values from chromatograms as a function of residence time and breakthrough percentage.

DBC_10_ measurements were performed using 2 mg/mL hIgG loading concentration at residence times (RT) of 12, 30 and 60 s. Figure 4 shows the chromatograms, and Figure 5 shows the calculated DBC_10_ values as functions of RT and breakthrough percentage. A *t*-test showed that there was no statistical significant in variation of DBC_10_ with flowrate. This phenomenon is common in membrane adsorbers, suggesting the convective transport of hIgG from solution to the Protein A ligand. Average DBC_10_ across all flowrates was 10.12 ± 1.82 mg/mL membrane.

Following saturation, the column was washed and hIgG was eluted from the column. The average elution volume across all residence times was 8.78 ± 0.55 MVs. The mass of hIgG eluted was measured and used to calculate a binding capacity. Assuming 100% recovery, this binding capacity also represents the EBC. The average EBC measured by this approach across all residence times was 29.51 ± 0.35 mg/mL membrane. This value was the same as q_max_ measured by batch adsorption at the 95% confidence level.

## 4. Discussion

This study demonstrated the preparation of Protein A affinity membranes using copper-free DBCO-azide click chemistry. Performance of these Protein A membranes, in terms of binding capacity, can be compared to other Protein A membranes described in the literature and to those that are commercially available. In 2015, Akashi et al. prepared Protein A membranes by immobilizing Protein A onto acrylic acid-grafted PVDF and PES membranes using EDC/Sulfo-NHS chemistry [13]. The resulting membranes had static binding capacities ranging from 0.55 to 0.75 mg IgG/mL membrane [13]. In 2009, Ma et al. prepared Protein A membrane adsorbers by immobilizing Protein A onto electrospun PES supports using EDC/NHS ester chemistry [12]. The resulting membranes showed a SBC of 4.5 mg IgG/mL membrane [12]. In 2008, Boi et al. reported the performance of a new Protein A membrane adsorber with SBC of 11.67 mg IgG/mL membrane and DBC_10_ of 5.76 mg IgG/mL membrane [17]. The preparation procedure for the membrane adsorber was not reported [17].

Commercially, up until 2017, the only available Protein A membrane was the Sartorius Sartobind^®^ Protein A, which reported a DBC_10_ of 5.9 mg IgG/mL membrane [18]. Newer commercial Protein A membranes, Purilogics’ Purexa™ PrA and Cytiva’s Fibro™ PrismA, were evaluated by Osuofa and Husson and shown to have ~70 mg/mL DBC_10_ at 5 s residence time and ~90 mg/mL SBC for hIgG [19]. Sartorius’ new convecdiff Protein A membranes have reported DBC_10_ values of ~43 mg/mL at 12 s residence time [20]. For these newer membrane devices, the preparation procedures are proprietary. In this study, the Protein A membranes prepared using the new click chemistry approach have shown binding capacity like other Protein A membranes in the literature.

The apparent K_d_ value also was benchmarked against other Protein A membranes. Previously, we measured K_d_ values of 7.07 × 10^−2^ ± 1.8 × 10^−2^ mg/mL for Purilogics Purexa™ PrA and 7.63 × 10^−2^ mg/mL for Sartorius Sartobind^®^ Protein A membranes [19]. K_d_ values from Protein A resins have been reported to be between 4.5 × 10^−2^ and 1.20 × 10^−1^ mg/mL by Hahn et al. [21]. Pabst et al. [22] reported K_d_ for Protein A resins to be between 7.99 × 10^−3^ and 1.05 × 10^−2^ mg/mL, depending on the mAb used for SBC experiments. By comparison, the K_d_ value of 1.72 × 10^−1^ ± 4.03 × 10^−2^ mg/mL for membranes prepared in this study is relatively high. Since 1/K_d_ is a mathematical representation of the affinity between immobilized Protein A and hIgG in solution, this relatively high K_d_ value (indicating weaker interaction between hIgG and this membrane compared to other Protein A media) may be a result of conjugating Protein A with DBCO-PEG_5_ molecules, which may interfere with the Fc binding domain.

The measured ligand density using Lowry assay was 3.47 ± 0.53 mg DBCO-Protein A/mL membrane, which results in a molar ratio of 2.60 + 0.18 hIgG molecules per Protein A molecule. Although the K_d_ value may be affected by the conjugation of Protein A, the binding efficiency remains within the expected range from approximately 2.54 to 3.3 antibody molecules to one Protein A molecule [23]. Additionally, other factors that may contribute to the ratio of hIgG to Protein A include intra- and inter- ligand steric hindrances [23], spatial distribution of the ligand on the membrane surface and pores [24], and multipoint attachment of the ligand during covalent immobilization [10]. The support itself can play a role on the spatial distribution of the ligand on the membrane. We selected regenerated cellulose over such supports as polyvinylidene fluoride and polyethersulfone, as the density of native surface functional groups is significantly higher for regenerated cellulose. Indeed, In the case of PVDF and PES membranes, surface treatments are normally performed to increase the number of reactive groups prior to ligand immobilization.

EV values for membrane adsorbers typically are not reported in the literature, but the measured EV of 8.78 ± 0.55 MVs is similar to values that we have measured for research scale Protein A membrane adsorbers [19], which ranged from 0.8 to 8.96 MVs. For Protein A resins, Pabst et al. [22] found that EVs range from 1.8 to 3.8 CVs. Higher EVs for membrane adsorbers can be attributed to the larger dead volume in membrane devices.

## 5. Conclusions

This study demonstrated a novel approach to fabricating Protein A membrane adsorbers using DBCO-azide copper-free click chemistry. The synthetic pathway consisted of three main steps: bioconjugation of Protein A with a DBCO-PEG_5_ linker, preparation of an azide-functionalized membrane surface, and click reaction of DBCO-Protein A onto the membrane surface. Synthesized Protein A membranes showed flow independent binding capacity and elution volumes that are typical for membrane adsorbers. From an operation point-of-view, these new Protein A membranes can be utilized in a rapid cycling approach for purification of IgG and other Fc-based proteins. In addition to Protein A, this new synthetic approach can be used for the immobilization of other ligands.

## Figures and Tables

**Table 1:** Molecular weight increments and corresponding number of DBCO-PEG_5_ groups conjugated to Protein A at different molar ratios of DBCO-PEG_5_-NHS ester to Protein A in solution.

Protein A Sample	Measured MW (g/mol)	MW Increase (g/mol)	#DBCO-PEG_5_ Added
Unconjugated	46,432 ± 91	-	-
7.5 to 1	47,150 ± 177	719 ± 152	1.2 ± 0.3
15 to 1	48,062 ± 226	1630 ± 207	2.8 ± 0.4
30 to 1	49,204 ± 148	2772 ± 117	4.8 ± 0.2

**Table 2:** XPS derived atomic composition of membranes modified using NH_2_-PEG_3_-N_3_ and DBCO-Protein A clicked membranes.

	C1s (%)	N1s (%)	O1s (%)
NH_2_-PEG_3_-N_3_ modified membrane	56.33 ± 0.37	6.34 ± 0.06	37.34 ± 0.30
DBCO-Protein A clicked membrane	60.60 ± 0.04	10.45 ± 0.53	28.95 ± 0.58

## Data Availability

The data required to reproduce these findings are available by request to the corresponding author.

## Supplementary Materials

The following supporting information can be downloaded at: https://www.mdpi.com/article/10.3390/membranes13100824/s1, Figure S1: Size exclusion chromatography (SEC) profile of DBCO-Protein A versus unconjugated Protein A.
