# Supplementary material for: Preparation of Protein A Membrane Adsorbers Using Strain-Promoted, Copper-Free Dibenzocyclooctyne (DBCO)-Azide Click Chemistry
Source: Membranes (Basel). 2023 Oct 6;13(10):824. doi: 10.3390/membranes13100824 (PMC10608826; doi:10.3390/membranes13100824)
Supplement: Supplementary file 1 [file membranes-13-00824-s001.zip › membranes-2533217-supplementary.pdf]

membranes

Article

Preparation of Protein A Membrane Adsorbers using Strain-Promoted, Copper-Free Dibenzocyclooctyne (DBCO)-Azide Click Chemistry

Joshua Osuofa, Scott Husson

Supplementary Materials:

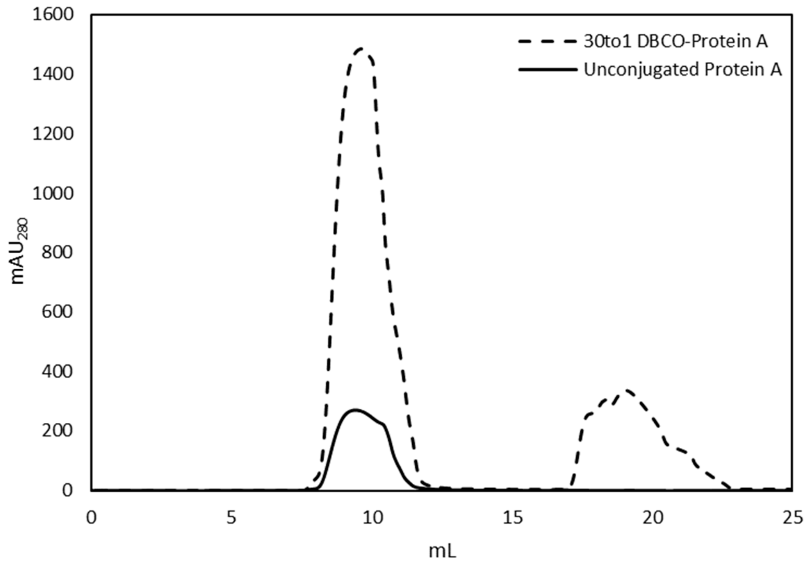

Figure S1. Size exclusion chromatography (SEC) profile of DBCO-Protein A versus unconjugated Protein A. One milliliter of 10 mg/mL sample was injected in both cases. The first peak corresponds to Protein A and the second peak corresponds to excess DBCO-PEG_5-NHS ester linker.
